# Cost-Effectiveness and Cost-Utility of Internet-Based Computer Tailoring for Smoking Cessation

**DOI:** 10.2196/jmir.2059

**Published:** 2013-03-14

**Authors:** Eline Suzanne Smit, Silvia MAA Evers, Hein de Vries, Ciska Hoving

**Affiliations:** ^1^CAPHRI School for Public Health and Primary CareDepartment of Health PromotionMaastricht UniversityMaastrichtNetherlands; ^2^CAPHRI School for Public Health and Primary CareDepartment of Health Services ResearchMaastricht UniversityMaastrichtNetherlands

**Keywords:** randomized controlled trial, economic evaluation, smoking cessation, Internet, computer-tailoring, general practice

## Abstract

**Background:**

Although effective smoking cessation interventions exist, information is limited about their cost-effectiveness and cost-utility.

**Objective:**

To assess the cost-effectiveness and cost-utility of an Internet-based multiple computer-tailored smoking cessation program and tailored counseling by practice nurses working in Dutch general practices compared with an Internet-based multiple computer-tailored program only and care as usual.

**Methods:**

The economic evaluation was embedded in a randomized controlled trial, for which 91 practice nurses recruited 414 eligible smokers. Smokers were randomized to receive multiple tailoring and counseling (n=163), multiple tailoring only (n=132), or usual care (n=119). Self-reported cost and quality of life were assessed during a 12-month follow-up period. Prolonged abstinence and 24-hour and 7-day point prevalence abstinence were assessed at 12-month follow-up. The trial-based economic evaluation was conducted from a societal perspective. Uncertainty was accounted for by bootstrapping (1000 times) and sensitivity analyses.

**Results:**

No significant differences were found between the intervention arms with regard to baseline characteristics or effects on abstinence, quality of life, and addiction level. However, participants in the multiple tailoring and counseling group reported significantly more annual health care–related costs than participants in the usual care group. Cost-effectiveness analysis, using prolonged abstinence as the outcome measure, showed that the mere multiple computer-tailored program had the highest probability of being cost-effective. Compared with usual care, in this group €5100 had to be paid for each additional abstinent participant. With regard to cost-utility analyses, using quality of life as the outcome measure, usual care was probably most efficient.

**Conclusions:**

To our knowledge, this was the first study to determine the cost-effectiveness and cost-utility of an Internet-based smoking cessation program with and without counseling by a practice nurse. Although the Internet-based multiple computer-tailored program seemed to be the most cost-effective treatment, the cost-utility was probably highest for care as usual. However, to ease the interpretation of cost-effectiveness results, future research should aim at identifying an acceptable cutoff point for the willingness to pay per abstinent participant.

## Introduction

### Background

Smoking is the single most preventable cause of illness and premature death in the world and is an important risk factor for 6 of the 8 leading causes of death, including several types of cancer, cardiovascular diseases, and respiratory diseases [1]. Consequently, smoking is related to a reduced quality of life and places a burden of €4 to €7 billion on health care [2-4]. Thus, quitting smoking is important, not only to improve individual and population health, but also to reduce smoking-related health care costs.

Extensive evidence exists on the clinical effectiveness of behavioral interventions for smoking cessation [5-7]. Brief advice from a general practitioner is one of these effective smoking cessation interventions [8]. However, general practitioners and practice nurses often report a lack of time and skills to provide their patients with elaborate smoking cessation advice [9,10]. Another behavioral intervention that has proven to be effective in increasing smoking cessation rates up to 13 months is computer tailoring [5,7,11-16]. Computer tailoring entails the adaption of the content of an intervention to participants’ individual characteristics by using computer programs [17]. Most often, a questionnaire is used as a screening instrument [12,18,19]. The answers smokers provided on the questions in this screening instrument are accumulated into a large data file and are subsequently matched with relevant feedback messages that are ultimately combined into a tailored feedback letter. Tailored interventions are more effective in attracting and keeping the smoker’s attention [17,20], resulting in better processing of information [21]. A single tailored feedback message is successful in increasing cessation rates [15], but dynamically tailored feedback provided on multiple occasions can be even more effective [11,12,22]. Due to the automatic generation of the tailored feedback and the fact that computer-tailored interventions are increasingly delivered online [23,24], the integration of an Internet-based computer-tailored program in the general practice setting might limit the burden on health professionals and patients, reduce facility and administrative costs, and could potentially be time- and cost-saving. As a combination of effective interventions was expected to achieve higher abstinence rates than either of the 2 alone [25], our research team has developed a smoking cessation intervention consisting of Internet-based multiple computer tailoring and a single tailored counseling session by a practice nurse.

Despite the proven clinical effectiveness of behavioral smoking cessation interventions, information about their relative cost-effectiveness is limited. Previously, several cost-effective smoking cessation interventions have been developed [26-28]. However, the interventions studied all involved the use of smoking cessation medication and investigated the cost-effectiveness of referrals to intensive counseling combined with pharmacotherapy [27], reimbursement of smoking cessation support [28], or the smoking cessation drug varenicline [26]. With regard to behavioral smoking cessation interventions, a computer-based smoking cessation intervention for primary care professionals was successful in increasing abstinence rates and quality-adjusted life years (QALYs) among patients [29]. In addition, in response to a call in the *Journal of Medical Internet Research* for research to economically evaluate eHealth interventions [30], cost-effectiveness and cost-utility studies of Internet-based interventions aimed at alcohol reduction [31] and depressive symptom treatment [32,33] have been initiated. However, to our current knowledge, no such studies have yet been conducted concerning the cost-effectiveness and cost-utility of an Internet-based smoking cessation intervention.

Therefore, the objective of the present study was to compare the cost-effectiveness and cost-utility of (1) an Internet-based multiple computer-tailored smoking cessation program combined with a single tailored counseling session by a practice nurses, (2) only an Internet-based multiple computer-tailored smoking cessation program, and (3) care as usual, defined as practice nurses’ standard care regarding smoking cessation.

## Methods

### Design

Economic evaluation studies aim to determine the costs and effects associated with an intervention and to compare these with the costs and effects of other interventions and/or current practice [34]. They usually consist of 5 steps [35], which are listed in Textbox 1. For a more extensive and detailed description of each of these steps, refer to Multimedia Appendix 1.

**The 5 steps in economic evaluation studies.**

**Step 1. Identification of relevant costs and effects**

Based on a chosen perspective (eg, the health care perspective, health insurer perspective, or societal perspective), relevant costs and effects are identified.

**Step 2. Measurement of costs and effects**

Costs can be assessed prospectively by means of cost diaries, or retrospectively using cost questionnaires. Effects are usually assessed in terms of quality of life.

**Step 3. Valuation of measured costs and effects**

Health care and patient costs are usually valued in a monetary currency using manuals for cost analysis in health care research. Effects on quality of life are usually valued in QALYs gained or lost.

**Step 4. Calculation of a cost-effectiveness ratio**

When comparing two interventions, an incremental cost-effectiveness ratio (ICER) can be calculated: ICER=(C_i_–C_c_)/(E_i_–E_c_). When comparing more than 2 interventions, a net monetary benefit (NMB) should be calculated using the willingness to pay (WTP): NMB=(E_i_–E_c_)×WTP–(C_i_–C_c_). A description of both formulas can be found in Multimedia Appendix 1.

**Step 5. Uncertainty analysis**

To deal with the sampling uncertainty bootstrap analyses can be used, whereas a sensitivity analysis can be conducted to deal with uncertainty due to the assumptions made.

The present economic evaluation study was trial-based, embedded in a randomized controlled trial (RCT) that tested the effectiveness of an Internet-based multiple computer-tailored smoking cessation program and tailoring counseling by practice nurses. This 3-armed RCT was conducted among Dutch adult smokers and had a follow-up period of 12 months. From May 2009 to June 2010, 91 practice nurses working in different Dutch general practices throughout the Netherlands recruited smoking patients for participation in the RCT. To aid recruitment, several recruitment materials were provided (eg, desk displays, posters, and business cards). Smokers interested in participation could sign up for the study on the study website. There, information was provided about the objectives of the study, the randomization procedure, and the incentive provided when respondents completed all questionnaires (ie, a €10 gift voucher). When signing up, participants were able to choose their own username and password and were informed that no one but the research team would be able to retrieve these passwords. After providing informed consent, participants were randomized into 1 of the 2 intervention groups (multiple tailoring and counseling or multiple tailoring only) or into the usual care control group. Randomization took place at the participant level by means of a computer software randomization device.

The trial design was approved by the Medical Ethics Committee of Maastricht University and the University Hospital Maastricht (MEC 08-3-037; NL22692.068.08), and is registered with the Dutch Trial Register (NTR1351). A more detailed description of the study design has been published elsewhere [25].

### Participants

Participants were eligible for participation if they smoked, were motivated to quit within 6 months, were 18 years or older, and were able to read and understand Dutch sufficiently to read study materials and participate in the trial. Moreover, they had to have access to the Internet. This resulted in a total of 414 eligible smokers.

### The Interventions

**Figure 1:**
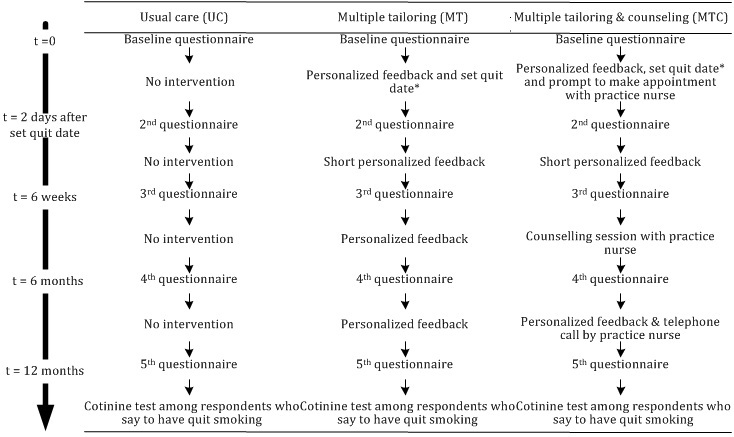
Overview of the intervention elements received by the 3 groups.

Figure 1 presents an overview of the intervention components in each of the study groups.

The Internet-based multiple computer-tailored smoking cessation program was based on a previously developed single computer-tailored intervention [12,15] for which the I-Change model (ICM) formed the theoretical framework [36]. As was its predecessor, the attitude-social influence-efficacy model [37], the ICM is a theory of behavioral change which incorporates theoretical concepts from several sociocognitive models, including the transtheoretical model [38], the theory of planned behavior [39], social cognitive theory [40], and the health belief model [41]. According to the ICM, the most proximal predictor of behavior is the intention to perform this behavior. Intention is predicted by 3 motivational constructs, attitude, perceived social influence, and self-efficacy, which in turn can be predicted by several premotivational factors, such as awareness, previous experience with the same and related behaviors, biological factors, and sociocultural factors. To overcome barriers that increase the well-known gap between intention and behavior (eg, [42]), the ICM proposes ability factors, such as an individual’s skills to refrain from smoking, and the formation of action plans. The ICM has been used successfully to develop several other effective computer-tailored programs [12,15,18]. While filling out the first online questionnaire (ie, baseline questionnaire), all participants were asked to set a date within the next 4 weeks at which they would attempt to quit smoking. They received a total of 4 feedback letters: at baseline, 2 days after the quit date they had set for themselves at baseline, after 6 weeks, and after 6 months. Feedback was personalized and tailored to several participant characteristics: gender, attitude, social influence and self-efficacy, intention to quit smoking, action planning, and smoking behavior. Feedback letters were iterative: the second, third, and fourth feedback letters did not only concern the participant’s present state, but also referred to changes participants had made since they were included in the program. Most feedback letters consisted of 4 to 5 pages and 7 components: (1) introduction, including specific feedback on the respondent’s smoking behavior and on his/her intention to quit smoking and to maintain nonsmoking; (2) feedback on the respondent’s attitude, including perceived advantages (pros) and disadvantages (cons) about smoking and quitting smoking; (3) feedback on perceived social influence (not) to smoke; (4) feedback on the respondent’s reported self-efficacy to refrain from smoking in specific situations, including suggestions on how to cope with these situations; (5) feedback on the extent to which respondents were planning to undertake specific actions (action plans) while preparing their quit attempt; (6) feedback on how to cope with situations in which it might be difficult not to smoke (coping plans), including the formulation of personal plans in the shape of if-then statements [28]; and (7) ending. Participants could access their feedback letters directly online after questionnaire completion. Additionally, feedback letters were sent to the participant by email. In both cases, feedback letters could be printed. An example of a tailored feedback message is provided in Multimedia Appendix 2.

After receiving the first tailored feedback, participants in the multiple tailoring and counseling group were prompted to schedule a counseling meeting with their practice nurse within 6 to 8 weeks. They received this counseling session instead of the third tailored feedback letter at the 6-week follow-up. A counseling protocol was provided to assist practice nurses in guiding these counseling sessions. This protocol consisted of 3 chapters guiding on 3 different types of participants: smokers who had quit successfully, smokers who had quit but relapsed, and smokers who had not yet quit. The content of the counseling session was developed to be as similar as possible to the content of the computer-tailored feedback and was also tailored to the participant characteristics mentioned previously. After 6 months, practice nurses were instructed to call their patients to ask them about their progress toward permanent cessation and, if needed, to provide them with additional cessation support.

Participants randomized in the usual care group received smoking cessation guidance according to participating practice nurses’ standard practice, which can vary from a brief intervention consisting of a single recommendation to stop smoking to more intensive interventions [43,44].

### Measurements

Self-reported online questionnaires were used to assess both costs and effects. Questionnaires were administered at baseline and at 6-week, 6-month, and 12-month follow-ups. When follow-up questionnaires were not completed by 1 week after the invitation, an email reminder was sent. At 12-month follow-up, this email reminder was followed by a phone call to collect data.

#### Identification, Measurement, and Valuation of Costs

The present economic evaluation study was conducted from a societal perspective. This implies that intervention costs, health care costs, and patient costs were identified as relevant. Intervention costs consisted of all costs that could be attributed to the delivery of the intervention, such as hosting costs for the Internet-based program and costs associated with counseling sessions. Costs for the development of the intervention and research-specific costs were excluded because these costs are sunk costs, costs that would already be spent before the intervention is implemented. In total, intervention costs were €57.70 per participant in the multiple tailoring and counseling group and €7.70 per participant in the multiple tailoring group. Interventions costs in the usual care group were considered zero because no intervention materials needed to be developed for this group. Health care costs related to general practitioners’ or practice nurses’ (telephone) consultations or home visits (other than the counseling session which was part of the multiple tailoring and counseling intervention), inpatient and outpatient specialist care, alternative medicine, mental health care, prescribed and over-the-counter (OTC) smoking cessation medication, hospital admissions, smoking cessation aids, and other care (eg, paramedics consultations or professional home care). Patient costs consisted of traveling and time lost due to participation in the intervention. However, for primary and secondary analyses, patient costs were not valued in monetary costs, but considered as reflected in participants’ reported quality of life [45].

Self-reported health care use was assessed during a 12-month follow-up period using a 3-month retrospective costing questionnaire that consisted of open-ended questions. Participants indicated whether they had received each type of care during the past 3 months, and if so, how often. The time participants spent using the online tailoring program was tracked by computer-registered log-in and log-out data. To assess time spent on counseling, we used a mean time of 20 minutes for face-to-face counseling sessions and an average of 10 minutes for telephone consultations. Traveling time was measured based on average travel distances to a general practitioner in the Netherlands [45].

To valuate health care usage and patient costs, the updated Dutch manual for cost analysis in health care research was used [45]. In general, standardized prices were used, but when no standardized prices were available, real costs or tariffs were used to estimate costs. In case of uncertainty, we used the lowest price. Costs of smoking cessation medication were calculated based on daily-defined dosage [46], including 6% Value Added Tax, prescription charges for prescribed medication, and clawback, a lawful discount percentage to be subtracted from medication prices by pharmacists [46]. Prices of informal care were based on shadow prices for unpaid work. The participants’ time spent on the program was valued by using the friction cost approach [45]. The index year used was 2011. Because prices in the Dutch manual for cost analysis in health care research [45] were from the year 2009, these prices were indexed to the year 2011. The consumer price indexes used were 105.38 for 2009 and 109.02 for 2011 [47]. A 13-month recruitment period and a 12-month follow-up period can both be considered as relatively short; therefore, it is unlikely that any substantial differences in health care consumption and effects existed between participants who were included at the beginning and toward the end of the recruitment period. As a result, there were no reasons to discount volumes of health care consumption or effects.

#### Identification, Measurement, and Valuation of Effects

The primary outcome measure used in the cost-effectiveness analysis (CEA) was prolonged abstinence measured at 12-month follow-up. This was assessed by 1 item asking whether the participant had refrained from smoking since the previous measurement at 6-month follow-up (ie, abstinence for at least 6 months; 1=no, 2=yes). Secondary outcome measures were 7-day point prevalence abstinence assessed by 1 item asking whether the participant had refrained from smoking during the past 7 days (1=no, 2=yes) and addiction level measured by the abbreviated Fagerström Test for Nicotine Dependence (FTND; 0=not addicted, 10=highly addicted) [48]. Self-reported abstinence at 12-month follow-up was cotinine validated using a saliva swab test [49]. Smoking abstinence was expressed in a probability score that a smoker would be abstinent (1=not abstinent, 2=abstinent).

The primary outcomes measure for the cost-utility analysis (CUA) was quality of life, measured in terms of QALYs. The valuation of effects on quality of life implies that utility scores need to be computed. In the present study, utilities were measured by the EuroQol EQ-5D [34,50], which is the current recommended measure for assessing quality of life by the National Institute for Health and Clinical Excellence [51] and has been used in other evaluations of smoking cessation programs [52,53] and Internet-based interventions aimed to reduce other health-related problems [32]. The EQ-5D consists of 5 health state dimensions (mobility, self-care, usual activity, pain/discomfort, and anxiety/depression) on which participants have to indicate their own health state (1=no complaints, 2=some complaints, 3=many complaints) [50]. Utility scores assessed at different points in time were transformed into an overall QALY score using the area under the curve method. The area under the curve is the duration of the health state (on the x-axis; in our case 12 months/1 year) multiplied by the quality weight for the health state (on the y-axis; utility scores). The resulting QALY score represents the number of QALYs gained or lost during the 12-month follow-up period [34]. For example, gaining 1 QALY means that 1 year is gained in perfect health, whereas gaining 0.8 QALY means that 1 year is gained in less than perfect health (utility score of 0.8) or that 0.8 years are gained in perfect health (utility score of 1).

### Analyses

All analyses were conducted according to the intention-to-treat principle. Missing data for costs, EQ-5D items, overall tobacco consumption, and addiction level were replaced by mean imputation by using participants’ scores on the previous and next measurement. When mean imputation was not possible because of missing data on multiple measurement points, missing data were replaced using the last observation carried forward (preferred choice) or next observation carried backward method. Missing data for smoking abstinence were replaced using a negative scenario; participants lost to follow-up were considered still smoking.

#### Baseline Comparability of the Three Study Groups

To investigate the comparability of the 3 groups with regard to demographics, baseline values of outcomes, and health care–related costs over the past 3 months, 1-way analyses of variance (ANOVA) with Tukey post hoc tests and chi-square tests were conducted. To determine whether selective dropout had occurred, a comparison was made between those lost to follow-up and those who remained in the study after 12 months using 2-sided *t* tests and chi-square tests.

#### Annual Costs and Effects

The 3 groups were compared with regard to their mean annual costs using nonparametric bootstrapping (5000 times) with 95% confidence intervals in percentiles [34]. To compare the 3 groups with regard to the mean effect assessed 12 months after baseline, 1-way ANOVA with Tukey post hoc tests and chi-square tests were conducted.

#### Cost-Effectiveness and Cost-Utility Analyses

First, incremental costs and effects were calculated for each of the 3 treatments studied. Subsequently, an incremental cost-effectiveness ratio (ICER) was calculated to compare costs and effects between pairs of study groups according to the following formula: ICER=(C_i_–C_c_)/(E_i_–E_c_). In this formula, *C*_*i*_ is the adjusted annual costs of the intervention group, *C*_*c*_ is the adjusted annual costs of the control group, *E*_*i*_ is the adjusted effects for the intervention group, and *E*_*c*_ is the adjusted effects of the control group. With regard to quality of life, ICERs are often called incremental cost-utility ratios (ICURs). However, because ratios such as the ICER or ICUR can compare only 2 groups, to compare the 3 groups in our study a net monetary benefit (NMB) was calculated for each of 3 treatments. The NMB can be calculated by valuing the effectiveness and utility outcomes in monetary values using a threshold for society’s willingness to pay (WTP) per abstinent participant and per QALY gained [54] according to the following formula: (E_i_–E_c_)×WTP–(C_i_–C_c_). In the present study, we used a WTP of €18,000 because this is an accepted Dutch cutoff point for the WTP per QALY [55], to calculate the likelihood that each treatment would be most likely highest in cost-effectiveness/cost-utility.

#### Uncertainty Analysis

Sampling uncertainty around the estimates of cost-effectiveness and cost-utility was taken into account by using nonparametric bootstrap resampling techniques [34,56]. Using bootstrapping techniques with replacement n (often 1000) times, a random sample is drawn from the original dataset resulting in 1000 slightly different samples and thus slightly different ICERs. Of these 1000 ICERs, the percentage can be calculated with (1) more effects and lower costs (dominant), (2) with less effects and lower costs, (3) with more effects and higher costs, and (4) with less effects and higher costs (inferior).

To deal with the uncertainty of parameter estimates from the primary analyses, a sensitivity analysis was conducted. As described earlier, in primary analyses patient costs (ie, traveling and time costs) were not valued in monetary costs but considered as reflected in participants’ reported quality of life [45]. However, because patient costs can be considered directly related to the treatment received [45], it could be argued that these costs should be included as part of the program cost. Therefore, we tested whether an increase in program costs as a result of patient costs’ monetary valuation would lead to a change in results. For the multiple tailoring and counseling group, the inclusion of patient costs meant an increase in program costs from €57.70 to €141.89 per participant; for the multiple tailoring group, this meant an increase in program costs from €7.70 to €82.24 per participant.

Bootstrap analyses were conducted using Microsoft Office Excel 2003. All other analyses were conducted using SPSS 17.0 (SPSS Inc, Chicago, IL, USA).

## Results

### Sample Characteristics

Of the 414 participants who were eligible for participation, 163 were randomized into the multiple tailoring and counseling group, 132 into the multiple tailoring group, and 119 into the usual care group. No baseline differences were found between the 3 groups (Table 1). After 12 months, 231 (55.8%) of the 414 participants could be followed up. Participants lost to follow-up were significantly younger than those who remained in the study (*P*=.01). Of the 46 participants who reported prolonged abstinence at the 12-month follow-up, 30 successfully completed a cotinine test and all cases of self-reported abstinence were confirmed. After imputation of missing values, total cost data were available for 370 participants (89.2%) whereas effect data were available for 414 (100%) participants for abstinence measures, 409 (98.8%) for addiction level, and 384 (92.8%) for QALYs.

### Annual Costs and Effects

A significant difference was found between the multiple tailoring and counseling and the usual care groups with regard to annual health care–related costs per patient, with significantly higher costs in the multiple tailoring and counseling group (Table 2). No differences were found between the 3 groups with regard to any specific type of health care–related costs (Table 2), nor regarding effects on abstinence, QALYs, or addiction level assessed at 12-month follow-up (Table 3).

**Table 1:** Comparability of the 3 groups, multiple tailoring and counseling (MTC), multiple tailoring (MT), and usual care (UC), regarding demographics, baseline values of outcomes, and health care–related costs over the past 3 months (N=414).

Variable		MTC (n=163)	MT (n=132)	UC (n=119)	*F* (df)	χ^2^ (df)	*P*
Age, mean (SD)		48.1 (12.0)	47.8 (12.5)	48.1 (11.3)	0.03 (2,406)		.97
Male, n (%)		60 (36.8)	41.2 (54)	42.9 (51)		1.2 (2)	.56
**Educational level, n (%)**						1.9 (2)	.76
	High	39 (23.9)	30 (22.9)	24 (20.2)			
	Medium	68 (41.7)	63 (48.1)	56 (47.1)			
	Low	56 (34.4)	38 (29.0)	39 (32.8)			
**Chronic diseases**							
	Cardiovascular diseases, n (%)	27 (16.6)	17 (13.0)	18 (15.1)		0.7 (2)	.69
	Respiratory diseases, n (%)	38 (23.3)	44 (33.6)	36 (30.3)		4.0 (2)	.14
	Diabetes, n (%)	8 (4.9)	6 (4.6)	7 (5.9)		0.2 (2)	.89
	Cancer, n (%)	10 (6.1)	12 (9.2)	8 (6.7)		1.1 (2)	.59
Cigarettes smoked per day, mean (SD)		20.6 (10.3)	23.5 (23.2)	21.5 (15.5)	1.14 (2,411)		.32
FTND^a^ score (range 0-10), mean (SD)		5.3 (2.2)	5.6 (2.0)	5.3 (2.1)	0.94 (2,406)		.39
Utility, mean (SD)^b^		0.8 (0.2)	0.8 (0.2)	0.8 (0.2)	0.60 (2,375)		.55
**Health care–related costs (€),** ^**c**^ **mean (SD)**		425.9 (1506.9)	286.9 (436.6)	236.9 (474.0)	1.19 (2,369)		.31
	General practitioner	53.2 (50.2)	61.0 (73.2)	49.7 (55.1)	1.11 (2,380)		.33
	Medical specialist	65.4 (132.1)	78.6 (170.9)	87.6 (202.8)	0.57 (2,373)		.57
	Hospital	206.9 (1371.3)	50.9 (205.2)	47.9 (230.8)	1.47 (2,378)		.23
	Alternative healer	5.1 (25.1)	9.4 (41.6)	4.9 (24.2)	0.84 (2,379)		.44
	Mental health care	30.3 (133.5)	24.3 (100.8)	38.2 (186.5)	0.27 (2,380)		.76
	Prescribed and OTC medication	26.7 (78.8)	36.4 (95.5)	13.6 (58.4)	2.37 (2,381)		.10
	Medical aids and assistive devices	1.7 (4.1)	3.4 (12.6)	1.9 (4.8)	1.73 (2,380)		.18
	Other care	26.0 (268.3)	19.2 (166.4)	12.5 (65.7)	0.15 (2,379)		.86

^a^ Fagerström Test for Nicotine Dependence (0=not addicted, 10=highly addicted)

^b^ Based on the Dutch algorithm for the EQ-5D scores.

^c^ Costs for prior 3 months.

**Table 2:** Mean annual costs^a^ per participant in the MTC, MT, and UC groups.

Cost type		Costs per group (€) mean (SD)^b^			95% CI^b^			
		MTC	MT	UC	MTC–MT		UC–MT	MTC–UC	
**Fixed costs**								
	Intervention costs (n=384)	57.70	7.70	0				
**Health care–related costs**								
	General practitioner (n=384)	157 (14)	180 (27)	139 (17)	–86.1 to 32.3		–105.8 to 15.5	–25.4 to 61.4
	Medical specialist (n=374)	298 (52)	251 (62)	224 (48)	–115.8 to 198.1		–188.3 to 116.6	–61.2 to 213.8
	Hospital (n=380)	610 (288)	267 (106)	172 (84)	–139.7 to 1054.7		–374.0 to 161.1	–17.0 to 1133.4
	Alternative healer (n=382)	17 (6)	29 (13)	18 (9)	–42.9 to 13.7		–43.1 to 18.6	–23.4 to 18.8
	Mental health care (n=384)	106 (39)	95 (34)	131 (71)	–92.2 to 109.8		–97.1 to 209.4	–200.2 to 111.9
	Prescribed and OTC smoking cessation medication (n=384)	148 (24)	144 (30)	90 (23)	–72.7 to 79.1		–129.6 to 18.5	–9.0 to 124.6
	Smoking cessation aids (n=384)	20 (10)	15 (10)	19 (14)	–21.6 to 32.5		–27.9 to 41.0	–34.4 to 32.3
	Other care (n=382)	122 (87)	21 (12)	41 (22)	–15.9 to 293.6		–24.7 to 72.4	–45.9 to 281.7
	Overall health care–related costs (n=370)	1564 (338)	1016 (158)	761 (122)	–95.4 to 1381.4		–642.2 to 139.1	194.3-1611.8

^a^ Volumes and price details are available upon request.

^b^ Based on 5000 bootstrap replications.

**Table 3:** Mean annual effect on smoking abstinence, QALY, and addiction level in the MTC, MT, and UC groups.

Effects	MTC	MT	UC	*F* (df)	χ^2^ (df)	*P*
Prolonged abstinent (n=414), n (%)	14 (8.6)	20 (15.2)	12 (10.1)		3.4 (2)	.19
QALY (EQ-5D)^a^ (n=384), mean (SD)	0.86 (0.15)	0.83 (0.21)	0.84 (0.21)	0.89 (2,381)		.41
7 days abstinent (n=414), n (%)	20 (12.3)	27 (20.5)	15 (12.6)		4.6 (2)	.10
FTND^b^ score (n=409), mean (SD)	4.76 (2.41)	5.21 (2.30)	4.81 (2.46)	1.40 (2,406)		.25

^a^ Based on the Dutch algorithm for the EQ-5D scores.

^b^ Fagerström Test for Nicotine Dependence (0=not addicted, 10=highly addicted); reversed range.

### Cost-Effectiveness Analyses

Table 4 shows that for participants in the multiple tailoring and counseling group costs were higher, whereas effects were lower than in the usual care and multiple tailoring groups; thus, multiple tailoring and counseling was dominated by the other 2 treatments. Comparing multiple tailoring with usual care showed that multiple tailoring resulted in more costs, but also in more effects. Compared with usual care, €5100 had to be paid within the multiple tailoring group for each additional abstinent participant (Table 4).

**Table 4:** Incremental costs and effects per abstinent smoker and per QALY gained for the MTC, MT, and UC groups with a willingness-to-pay threshold of €18,000.

Intervention		Incremental costs (€)	Incremental probability^a^	Incremental costs^b^ (€)
**Prolonged abstinence** ^**c**^				
	UC			
	MT vs UC	255	.05	5100
	MTC vs UC	806	–.02	Dominated^d^
	MTC vs MT	551	–.07	Dominated^e^
**QALY (EQ-5D)** ^**f**^				
	UC			
	MT vs UC	255	–.01	Dominated^g^
	MTC vs UC	806	.02	40,300
	MTC vs MT	551	.03	18,367

^a^ Probability of being abstinent/gaining 1 QALY.

^b^ Per abstinent participant or per QALY; calculated according to the formula ICER/ICUR=(C_i_–C_c_)/(E_i_–E_c_); additional information available in Multimedia Appendix 1.

^c^ Coded as 1=not abstinent and 2=abstinent.

^d^ ICER=–40.300.

^e^ ICER=–7.871.

^f^ Based on the Dutch algorithm for the EQ-5D scores.

^g^ ICUR=–25.500.

**Figure 2:**
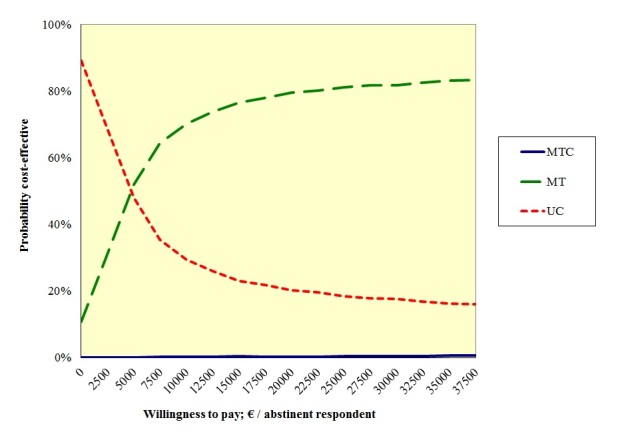
Cost-effectiveness acceptability curve for the 3 treatments studied: MTC, MT, and UC.

The CEA showed that until a threshold value for the WTP of €5100 per abstinent participant, usual care was probably the most efficient treatment. However, from a WTP of €5100 or higher, multiple tailoring was probably most cost-effective (Table 5). With the accepted Dutch cutoff point of €18,000 per QALY for preventive interventions [57], multiple tailoring would be the preferable treatment. These results are visually displayed in the cost-effectiveness acceptability curve (CEAC), showing the probability of each treatment being preferable to the other 2 treatments for varying levels of the WTP per additional abstinent participant (Figure 2). Sensitivity analyses supported these results (Table 5).

Results from secondary analyses showed that with 7-day point prevalence abstinence, a high probability was found (ie, 88%, with a WTP of €18,000 per abstinent participant) that multiple tailoring was the most cost-effective treatment. Regarding the level of addiction, however, it was most probable that multiple tailoring would be least efficient (Table 5).

### Cost-Utility Analyses

With regard to QALYs gained, Table 4 shows that multiple tailoring was dominated by usual care because this treatment was both more expensive and less effective. Furthermore, cost-utility analyses showed that multiple tailoring and counseling was more expensive, but also more effective than usual care and multiple tailoring in increasing the number of QALYs gained. This resulted in an incremental cost of €40,300 per QALY gained when comparing multiple tailoring and counseling with usual care, and in an incremental cost of €18,367 per QALY when comparing multiple tailoring and counseling to multiple tailoring.

**Figure 3:**
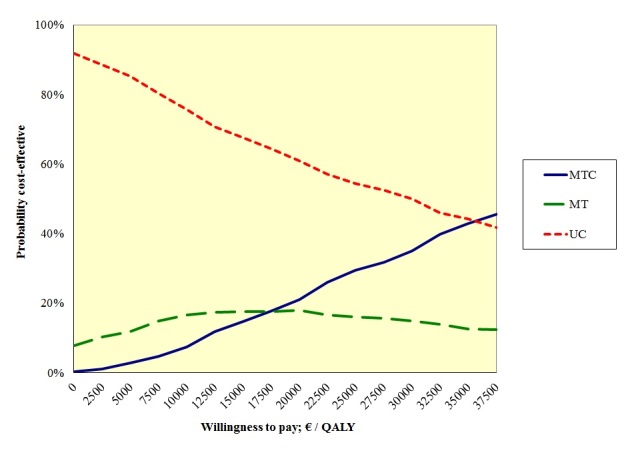
Cost-utility acceptability curve for the 3 treatments studied: MTC, MT, and UC.

With a WTP of €18,000 per abstinent participant, the CUA showed that usual care would probably (ie, 64%) be the most efficient treatment (Table 5). Although decreasing this threshold value to €0 led to an increased probability that usual care would be most efficient, increasing this threshold led to a lower probability of usual care being most preferable. With a WTP of almost €40,000, usual care and multiple tailoring and counseling would be equally preferable. These results are further illustrated in the cost-utility acceptability curve (CUAC) (Figure 3). Sensitivity analyses showed similar results (Table 5).

**Table 5:** Results from cost-effectiveness and cost-utility analyses based on 1000 bootstrap replications.

Type of analysis		Group, n			Probability of highest net monetary benefit^a^, %		
		MTC	MT	UC	MTC	MT	UC
**Primary analysis**							
	Prolonged abstinence^f^	145	121	104	0	78	21
	QALY (EQ-5D)^b^	145	121	104	18	18	64
**Secondary analysis**							
	7-day ppa^c,f^	145	121	104	1	88	11
	FTND score^d^	135	115	96	50	6	45
**Sensitivity analysis** ^**e**^							
	Prolonged abstinence^f^	145	121	104	1	76	24
	QALY (EQ-5D)^b^	145	121	104	19	15	66

^a^With a willingness-to-pay threshold of €18,000.

^b^Based on the Dutch algorithm for the EQ-5D scores.

^c^ppa: point prevalence abstinence.

^d^FTND: Fagerström Test for Nicotine Dependence (0=not addicted, 10=highly addicted).

^e^Increase in program costs from €57.70 to €141.89 (MTC group) and from €7.70 to €82.24 (MT group) caused by the inclusion of patient costs.

^f^Coded as 1=not abstinent and 2=abstinent.

## Discussion

### Main Findings

To our current knowledge, this was the first study to determine the cost-effectiveness and cost-utility of a behavioral smoking cessation intervention consisting of Internet-based computer tailoring with and without counseling by a practice nurse. The results presented suggest that participants who received the Internet-based multiple computer-tailored program and tailored counseling by their practice nurse reported significantly more annual health care-related costs than participants who received care as usual. A potential explanation for this finding might be that smokers were prompted by the tailored feedback they received to ask for more smoking cessation guidance, eg, additional counseling sessions with the practice nurse or a prescription for smoking cessation medication. Although participants who received the Internet-based program only might have had the same tendency, the practice nurses for participants in the multiple tailoring and counseling group might have been prompted by their patients’ visit to offer them more smoking cessation help. Because the current smoking cessation guidelines in the Netherlands recommend more than 1 counseling session [44,58], this is not unlikely. Interestingly, though the significant difference in total health care-related costs could not be explained by a difference in any particular type of health care-related costs. For instance, no significant differences were found with regard to costs spent on general practitioner or practice nurse consultations or on the use of smoking cessation aids.

Furthermore, the present study showed that the Internet-based multiple computer-tailored smoking cessation program would probably be the most cost-effective of the 3 treatments under study. Although no similar studies yet exist within the field of smoking cessation, this finding is in-line with findings from recent studies toward the cost-effectiveness of Internet-based interventions aimed at other health-related behaviors or health problems [31,32]. Compared with current practice, the incremental costs per abstinent participant associated with the Internet-based multiple computer-tailored smoking cessation program were €5100. This is slightly more than what was found in previous studies [59,60]. However, one of these studies only included costs directly related to the interventions received [59], whereas we conducted our economic evaluation from a broader societal perspective. Nevertheless, the interpretation of the incremental costs per abstinent participant is difficult because no information exists on the amount of money that society is willing to pay per abstinent participant. Although a WTP of €18,000 per QALY is an accepted Dutch cutoff point [55], no such cutoff point exists with regard to smoking abstinence rates. To enable the interpretation of the incremental costs per abstinent participant, future research should aim at identifying an acceptable cutoff point for the WTP per abstinent participant.

Regarding cost-utilities, the results suggest that care as usual would probably be the most preferable of the treatments studied. A potential explanation for this finding might be that the follow-up period of 12 months was not sufficiently long for the beneficial effects of the intervention on smoking abstinence to be translated into detectable changes in quality of life, as recent ex-smokers are known to suffer from withdrawal symptoms [61]. A potential solution would be to use short-term trial data as input for a model predicting the effects of smoking cessation interventions on long-term quality of life, a technique used in several recent studies [28,29,60]. Although trial data served as input for these models, several assumptions had to be made to build these models, bringing about additional uncertainty in the results presented [34, 60]. Another solution would be to lengthen the follow-up period of clinical trials to gather longer-term data on quality of life. Although this might imply an increased burden on the participant, it might be needed to establish the intervention’s cost-utility in an as certain as possible way. However, as previous studies were able to detect a positive association between quitting smoking and quality of life during a 12-month follow-up period [62,63], additional explanations for this finding need to be sought. One such explanation might be that within Dutch general practices, care as usual for smoking cessation is rather intensive. Anecdotal evidence suggests that practice nurses usually offer 4 to 6 consultations as part of smoking cessation care (unpublished). Although participants in the multiple tailoring and multiple tailoring and counseling groups received 4 and 3 tailored feedback letters, respectively, a primarily Internet-based program might have been perceived as more distant and less intense than care as usual. As a result, participants in the usual care group might have established a better or stronger social bond with their practice nurse than participants in the multiple tailoring or multiple tailoring and counseling groups. This assumed social bond [64] might subsequently have resulted in positive effects on quality of life among usual care participants.

### Strengths and Limitations

The present study aimed to contribute to the literature by examining the cost-effectiveness and cost-utility of an Internet-based smoking cessation intervention, something that has not been done before to our knowledge. In the present study, in addition to generic quality of life, disease-specific effects of the intervention (ie, smoking abstinence) were taken into account. To facilitate the comparison of the cost-effectiveness of interventions targeting different diseases, effects are usually assessed in terms of quality of life. However, to compare smoking cessation interventions more specifically, disease-specific effect measures might be more informative.

Nevertheless, the present study also had its limitations. First, it suffered from relatively high dropout rates. High rates of attrition seem to be inherent to many Internet-based interventions and dropout rates of 44% are not uncommon [24,65-67]. As a consequence, however, there was not sufficient power for us to conduct a complete-case analysis as part of the sensitivity analyses. Secondly, because we expected higher attrition rates in the multiple tailoring and counseling group, slightly more participants were randomized into this intervention group at baseline. However, attrition rates appeared to be similar among the groups, resulting in a skewed distribution of participants with 163 participants in the multiple tailoring and counseling group, 132 in the multiple tailoring group, and 119 in the usual care group. Although we do not expect that this has biased our results, this finding of no selective attrition can be considered valuable in the design of future trials.

### Conclusions

The Internet-based multiple computer-tailored program seemed to be the most cost-effective treatment when smoking abstinence was the outcome measure. However, the cost-utility, using quality of life as the outcome measure, was probably the highest with care as usual. To enable the interpretation of the incremental costs per abstinent participant found in the cost-effectiveness analyses, future research should aim at identifying an acceptable cutoff point for the WTP per abstinent participant.

## Multimedia Appendix 1

Economic evaluation studies in a nutshell.

## Multimedia Appendix 2

A tailored smoking cessation advice for a respondent who reported to still be smoking at six-month follow-up and whose self-efficacy to quit has decreased since baseline.

## Multimedia Appendix 3

CONSORT-EHEALTH checklist V1.6.2 [68].

## Abbreviations

CEA: cost-effectiveness analysis
CUA: cost-utility analysis
CEAC: cost-effectiveness acceptability curve
CUAC: cost-utility acceptability curve
FTND: Fagerström Test for Nicotine Dependence
ICER: incremental cost-effectiveness ratio
ICM: I-Change Model
ICUR: incremental cost-utility ratio
MTC: multiple tailoring and counseling
MT: multiple tailoring
NMB: net monetary benefit
OCT: over-the-counter
QALY: quality-adjusted life year
RCT: randomized controlled trial
UC: usual care
WTP: willingness to pay

## References

[1] (2008). WHO Report on the Global Tobacco Epidemic, 2008: the MPOWER package.

[2] Allender S, Balakrishnan R, Scarborough P, Webster P, Rayner M (2009). The burden of smoking-related ill health in the UK. Tob Control.

[3] Muennig P, Fiscella K, Tancredi D, Franks P (2010). The relative health burden of selected social and behavioral risk factors in the United States: implications for policy. Am J Public Health.

[4] Scarborough P, Bhatnagar P, Wickramasinghe KK, Allender S, Foster C, Rayner M (2011). The economic burden of ill health due to diet, physical inactivity, smoking, alcohol and obesity in the UK: an update to 2006-07 NHS costs. J Public Health (Oxf).

[5] Lancaster T, Stead LF (2005). Self-help interventions for smoking cessation. Cochrane Database Syst Rev.

[6] Lancaster T, Stead LF (2005). Individual behavioural counselling for smoking cessation. Cochrane Database Syst Rev.

[7] Noar SM, Benac CN, Harris MS (2007). Does tailoring matter? Meta-analytic review of tailored print health behavior change interventions. Psychol Bull.

[8] Stead LF, Bergson G, Lancaster T (2008). Physician advice for smoking cessation. Cochrane Database Syst Rev.

[9] Hall S, Vogt F, Marteau TM (2005). A short report: survey of practice nurses' attitudes towards giving smoking cessation advice. Fam Pract.

[10] Vogt F, Hall S, Marteau TM (2005). General practitioners' and family physicians' negative beliefs and attitudes towards discussing smoking cessation with patients: a systematic review. Addiction.

[11] Borland R, Balmford J, Hunt D (2004). The effectiveness of personally tailored computer-generated advice letters for smoking cessation. Addiction.

[12] Dijkstra A, De Vries H, Roijackers J (1998). Long-term effectiveness of computer-generated tailored feedback in smoking cessation. Health Educ Res.

[13] Hoving C, Mudde AN, Dijk F, Vries HD (2010). Effectiveness of a smoking cessation intervention in Dutch pharmacies and general practices. Health Education.

[14] Strecher VJ (1999). Computer-tailored smoking cessation materials: a review and discussion. Patient Educ Couns.

[15] Te Poel F, Bolman C, Reubsaet A, de Vries H (2009). Efficacy of a single computer-tailored e-mail for smoking cessation: results after 6 months. Health Educ Res.

[16] Rabius V, Pike KJ, Wiatrek D, McAlister AL (2008). Comparing internet assistance for smoking cessation: 13-month follow-up of a six-arm randomized controlled trial. J Med Internet Res.

[17] de Vries H, Brug J (1999). Computer-tailored interventions motivating people to adopt health promoting behaviours: introduction to a new approach. Patient Educ Couns.

[18] van Keulen HM, Mesters I, Brug J, Ausems M, Campbell M, Resnicow K, Zwietering PJ, van Breukelen G, van Mechelen W, Severens JL, de Vries H (2008). Vitalum study design: RCT evaluating the efficacy of tailored print communication and telephone motivational interviewing on multiple health behaviors. BMC Public Health.

[19] van Stralen MM, Kok G, de Vries H, Mudde AN, Bolman C, Lechner L (2008). The Active plus protocol: systematic development of two theory- and evidence-based tailored physical activity interventions for the over-fifties. BMC Public Health.

[20] Kreuter M (2000). The Active plus protocol: systematic development of two theory- and evidence-based tailored physical activity interventions for the over-fifties. Tailoring Health Messages: Customizing Communication with Computer Technology.

[21] Dijkstra A (2005). Working mechanisms of computer-tailored health education: evidence from smoking cessation. Health Educ Res.

[22] Dijkstra A, De Vries H, Roijackers J, van Breukelen G (1998). Tailoring information to enhance quitting in smokers with low motivation to quit: three basic efficacy questions. Health Psychol.

[23] Lustria ML, Cortese J, Noar SM, Glueckauf RL (2009). Computer-tailored health interventions delivered over the Web: review and analysis of key components. Patient Educ Couns.

[24] Shahab L, McEwen A (2009). Online support for smoking cessation: a systematic review of the literature. Addiction.

[25] Smit ES, de Vries H, Hoving C (2010). The PAS study: a randomized controlled trial evaluating the effectiveness of a web-based multiple tailored smoking cessation programme and tailored counselling by practice nurses. Contemp Clin Trials.

[26] Annemans L, Nackaerts K, Bartsch P, Prignot J, Marbaix S (2009). Cost effectiveness of varenicline in Belgium, compared with bupropion, nicotine replacement therapy, brief counselling and unaided smoking cessation: a BENESCO Markov cost-effectiveness analysis. Clin Drug Investig.

[27] Hoogendoorn M, Feenstra TL, Hoogenveen RT, Rutten-van Mölken MP (2010). Long-term effectiveness and cost-effectiveness of smoking cessation interventions in patients with COPD. Thorax.

[28] Vemer P, Rutten-van Mölken MP, Kaper J, Hoogenveen RT, van Schayck CP, Feenstra TL (2010). If you try to stop smoking, should we pay for it? The cost-utility of reimbursing smoking cessation support in the Netherlands. Addiction.

[29] Smith MY, Cromwell J, DePue J, Spring B, Redd W, Unrod M (2007). Determining the cost-effectiveness of a computer-based smoking cessation intervention in primary care. Manag Care.

[30] Ahern DK, Kreslake JM, Phalen JM (2006). What is eHealth (6): perspectives on the evolution of eHealth research. J Med Internet Res.

[31] Smit F, Lokkerbol J, Riper H, Majo MC, Boon B, Blankers M (2011). Modeling the cost-effectiveness of health care systems for alcohol use disorders: how implementation of eHealth interventions improves cost-effectiveness. J Med Internet Res.

[32] Warmerdam L, Smit F, van Straten A, Riper H, Cuijpers P (2010). Cost-utility and cost-effectiveness of internet-based treatment for adults with depressive symptoms: randomized trial. J Med Internet Res.

[33] Gerhards SA, de Graaf LE, Jacobs LE, Severens JL, Huibers MJ, Arntz A, Riper H, Widdershoven G, Metsemakers JF, Evers SM (2010). Economic evaluation of online computerised cognitive-behavioural therapy without support for depression in primary care: randomised trial. Br J Psychiatry.

[34] Drummond MF, Sculpher MJ, Torrance G, O'Brien B, Stoddart G (2005). Methods for the economic evaluation of health care programmes.

[35] Evers SM, Wolf C, van Heugten C (2010). Economische evaluatie van neuropsychologische behandeling. Neuropsychologische Behandeling.

[36] de Vries H, Mudde AN, Leijs I, Charlton A, Vartiainen E, Buijs G, Clemente MP, Storm H, González Navarro A, Nebot M, Prins T, Kremers S (2003). The European Smoking Prevention Framework Approach (EFSA): an example of integral prevention. Health Educ Res.

[37] de Vries HD, Mudde AN (1998). Predicting stage transitions for smoking cessation applying the attitude-social influence-efficacy model. Psychology & Health.

[38] Prochaska JO, Redding CA, Evers KE, Glanz K (1997). The transtheoretical model and stages of change. Health Behavior and Health Education: Theory, Research, and Practice.

[39] Ajzen I (1985). From intentions to actions: a theory of Planned Behaviour. Action-Control: From Cognition to Behavior.

[40] Bandura A (1986). Social Foundations of Thought and Action: A Social Cognitive Theory.

[41] Janz NK, Champion VL, Strecher VJ, Glanz K (2002). The health belief model. Health Behavior and Health Education: Theory, Research, and Practice.

[42] Armitage CJ, Conner M (2001). Efficacy of the Theory of Planned Behaviour: a meta-analytic review. Br J Soc Psychol.

[43] Chavannes NH, Kaper J, Frijling BD, Van der Laan JR, Jansen PWM, Guerrouj S, Drenthen AJM, Bax W, Wind LA (2009). NHG-Standaard Stoppen met roken. NHG-standaarden voor de huisarts.

[44] Partnership Stop met Roken (2009). Richtlijn Behandeling van Tabaksverslaving herziening 2009.

[45] Hakkaart-van Roijen L, Tan SS, Bouwmans CAM (2010). Handleiding voor kostenonderzoek: Methoden en standaard kostprijzen voor economische evaluaties in de gezondheidszorg. Geactualiseerde versie 2010.

[46] Commissie Farmacotherapautische Hulp CVZ (2011). Farmacotherapeutisch Kompas: medisch farmaceutische voorlichting.

[47] Centraal Bureau voor de Statistiek (2011). Consumentenprijzen; prijsindex 2006.

[48] Heatherton TF, Kozlowski LT, Frecker RC, Fagerström KO (1991). The Fagerström Test for Nicotine Dependence: a revision of the Fagerström Tolerance Questionnaire. Br J Addict.

[49] Craig Medical Distribution Inc.

[50] The EuroQol Group (1990). EuroQol--a new facility for the measurement of health-related quality of life. Health Policy.

[51] Sculpher M (2008). NICE's 2008 Methods Guide: sensible consolidation or opportunities missed?. Pharmacoeconomics.

[52] Hoogendoorn M, Welsing P, Rutten-van Mölken MP (2008). Cost-effectiveness of varenicline compared with bupropion, NRT, and nortriptyline for smoking cessation in the Netherlands. Curr Med Res Opin.

[53] Hoogwegt MT, Hoeks SE, Pedersen SS, Scholte op Reimer WJ, van Gestel YR, Verhagen HJ, Poldermans D (2010). Smoking cessation has no influence on quality of life in patients with peripheral arterial disease 5 years post-vascular surgery. Eur J Vasc Endovasc Surg.

[54] Stinnett AA, Mullahy J (1998). Net health benefits: a new framework for the analysis of uncertainty in cost-effectiveness analysis. Med Decis Making.

[55] Casparie AF, van Hout BA, Simoons ML (1998). [Guidelines and costs]. Ned Tijdschr Geneeskd.

[56] Glick HA, Doshi JA, Sonnad SS, Polsky D (2007). Economic Evaluation in Clinical Trials.

[57] Raad voor de Volksgezondheid en Zorg (2006). Zoetermeer.

[58] Pieterse ME, Seydel ER, DeVries H, Mudde AN, Kok GJ (2001). Effectiveness of a minimal contact smoking cessation program for Dutch general practitioners: a randomized controlled trial. Prev Med.

[59] Salize HJ, Merkel S, Reinhard I, Twardella D, Mann K, Brenner H (2009). Cost-effective primary care-based strategies to improve smoking cessation: more value for money. Arch Intern Med.

[60] Kaper J, Wagena EJ, van Schayck CP, Severens JL (2006). Encouraging smokers to quit: the cost effectiveness of reimbursing the costs of smoking cessation treatment. Pharmacoeconomics.

[61] Shiffman S, Patten C, Gwaltney C, Paty J, Gnys M, Kassel J, Hickcox M, Waters A, Balabanis M (2006). Natural history of nicotine withdrawal. Addiction.

[62] Hays JT, Croghan IT, Baker CL, Cappelleri JC, Bushmakin AG (2012). Changes in health-related quality of life with smoking cessation treatment. Eur J Public Health.

[63] Sales MP, Oliveira MI, Mattos IM, Viana CM, Pereira ED (2009). The impact of smoking cessation on patient quality of life. J Bras Pneumol.

[64] Shum C, Humphreys A, Wheeler D, Cochrane MA, Skoda S, Clement S (2000). Nurse management of patients with minor illnesses in general practice: multicentre, randomised controlled trial. BMJ.

[65] McKay HG, Danaher BG, Seeley JR, Lichtenstein E, Gau JM (2008). Comparing two web-based smoking cessation programs: randomized controlled trial. J Med Internet Res.

[66] Wangberg SC, Bergmo TS, Johnsen JA (2008). Adherence in Internet-based interventions. Patient Prefer Adherence.

[67] Wangberg SC, Nilsen O, Antypas K, Gram IT (2011). Effect of tailoring in an internet-based intervention for smoking cessation: randomized controlled trial. J Med Internet Res.

[68] Eysenbach G, CONSORT-EHEALTH Group (2011). CONSORT-EHEALTH: improving and standardizing evaluation reports of Web-based and mobile health interventions. J Med Internet Res.

