# Supplementary material for: Cost-Effectiveness and Cost-Utility of Internet-Based Computer Tailoring for Smoking Cessation
Source: J Med Internet Res. 2013 Mar 14;15(3):e57. doi: 10.2196/jmir.2059 (PMC3636293; doi:10.2196/jmir.2059)
Supplement: Supplementary file 1 [file jmir_v15i3e57_app1.pdf]

Multimedia Appendix 1. Economic evaluation studies in a nutshell

An economic evaluation study aims to determine the costs and effects associated with an intervention and to compare these with other interventions and/or current practice [1]. Results from economic evaluation studies can inform decisions concerning widespread implementation of these interventions, Economic evaluation studies usually consist of five steps [2]. In this multimedia appendix, a brief description is provided of each of these steps, in order to provide an overview for readers who are less familiar with this type of studies.

The first step consists of *identifying relevant costs and effects*, looking at the interventions compared and the disease under consideration. This decision is made in line with a chosen perspective, e.g. the health care perspective, health insurer perspective or the societal perspective. However, decision-makers usually base their decision to finance and/or implement an intervention on a large scale on all potential societal consequences, both within and outside the care setting. Economic evaluations are therefore preferably conducted from a societal perspective, in which all relevant costs and effects are taken into account [3].

The second step concerns the *measurement of costs and effects*. Costs can be assessed using medical records or self-reported measures. For instance, the disadvantage of medical records is that they limit their recoding to standard health care usage and are thus unsuitable as a sole source within a societal perspective, including both costs inside (i.e. standard health care costs) and outside (i.e. alternative health care costs, over-the-counter medication, patient costs) of the care setting. A recently conducted review that found high levels of agreement between self-reported cost data and registered cost data, even concluded that self-reported data were more reliable and valid [4]. Self-reported cost data can be collected prospectively by means of cost diaries or retrospectively using cost questionnaires. Again, however, both methods have advantages and disadvantages. Questionnaires, for instance, are by nature retrospective and are thus subject to recall error. On the other hand, the burden of filling out a prospective cost diary during a relatively long follow-up period could be rather high [5]. Effects are usually assessed in terms of quality of life. While both generic and disease-specific quality of life instruments exist,

decision-makers want to compare the cost-effectiveness of interventions targeting different diseases. Measuring generic quality of life is therefore usually preferable.

A valuation of the measured costs and effects takes place in the third step. With regard to costs this implies that the number of visits, days and hours are expressed in a monetary currency. To valuate health care and patient costs, manuals for cost analysis in health care research can be used [6]. These manuals include the standardized average cost price of health care facilities in a particular country. When no standardized cost prices are available, real costs or tariffs can be used to estimate costs. As it is important that all cost prices are comparable, all cost prices should be indexed to the same year. The valuation of effects on quality of life implies that, first, utility scores need to be computed. Utilities refer to preferences that individuals or society may have for a particular set of health outcomes and can range from 0 (the worst imaginable health state) to 1 (perfect health) [1]. Subsequently, utility scores assessed at different points in time can be transformed into an overall QALY score, representing the number of QALYs gained or lost during the follow-up period. Gaining one QALY means that one year is gained in perfect health, while e.g. gaining 0.8 QALY means that one year is gained in less than perfect health or that 0.8 years are gained in perfect health.

In the fourth step of the economic evaluation *a cost-effectiveness ratio* is calculated. In this step, costs and effects are brought together in a ratio. This incremental cost-effectiveness ratio (ICER) can be calculated according to the following formula: ICER = (Ci-Cc) / (Ei-Ec), where Ci are the adjusted annual costs of the intervention group, Cc the adjusted annual costs of the control group, Ei the adjusted effects for the intervention group and Ec the adjusted effects of the control group. With regard to quality of life, ICERs are often called ICURs: incremental cost-utility ratios. Ratio's such as the ICER or ICUR are, however, able to compare only two groups with each other. An approach which allows us to compared more than two groups with each other when choosing among treatments is to calculate a net monetary benefit (NMB) for each of the treatments. The NMB can be calculated by valuing the effectiveness and utility outcomes in monetary values using a threshold for society's willingness to pay (WTP) per abstinent participant and per QALY gained [7], according to the following formula: (Ei-Ec)*WTP - (Ci-Cc). If the NMB is above 0, the benefits outweigh the costs and the intervention is considered cost-effective. As many new interventions bring about both additional costs and additional

effects, cut-off points have been determined for the WTP per additional measure of effect. In the Netherlands, cut-off points have been established for the WTP per QALY, varying by the severity of the condition [8]. For preventive interventions, €18,000 per QALY has been established as an accepted Dutch cutoff point [8]. Similar cut-off points do not yet exist for more behavior-specific results of an intervention, such as abstinence rates.

The fifth and last step entails an *uncertainty analysis*. Each economic evaluation study can suffer from several types of uncertainty. A first type of uncertainty inherent to economic evaluations is sampling uncertainty. Accidentally, participants can be included in the economic evaluation who reported, for instance, very high health care costs. To deal with this type of uncertainty, bootstrap analyses can be conducted [1, 9]. Using bootstrapping techniques with replacement n (often 1000) times a random sample is drawn from the original dataset, resulting in 1000 slightly different samples and thus slightly different ICERs. Of these 1000 ICERs, the percentage can be calculated with 1) more effects and lower costs (dominant); 2) with less effects and lower costs; 3) with more effects and higher costs and 4) with less effects and higher costs (inferior). The percentages of ICERs falling within each of these four categories can be visually displayed in a cost-effectiveness plane. A simplified example of such a cost-effectiveness plane is presented in figure 1. Subsequently, in a cost-effectiveness acceptability curve (CEAC), the probability of each treatment being most preferable compared with the other treatment(s) studied can be shown for varying levels of the WTP per additional unit of effect [7], i.e. per additional abstinent participant or per additional QALY gained (for examples, see figures 2 and 3 in the main article). Secondly, uncertainty results from the fact that for an economic evaluation study it is rather hard to obtain precisely correct data and that certain assumptions have to be made. To test whether results are robust, a sensitivity analysis can be conducted.

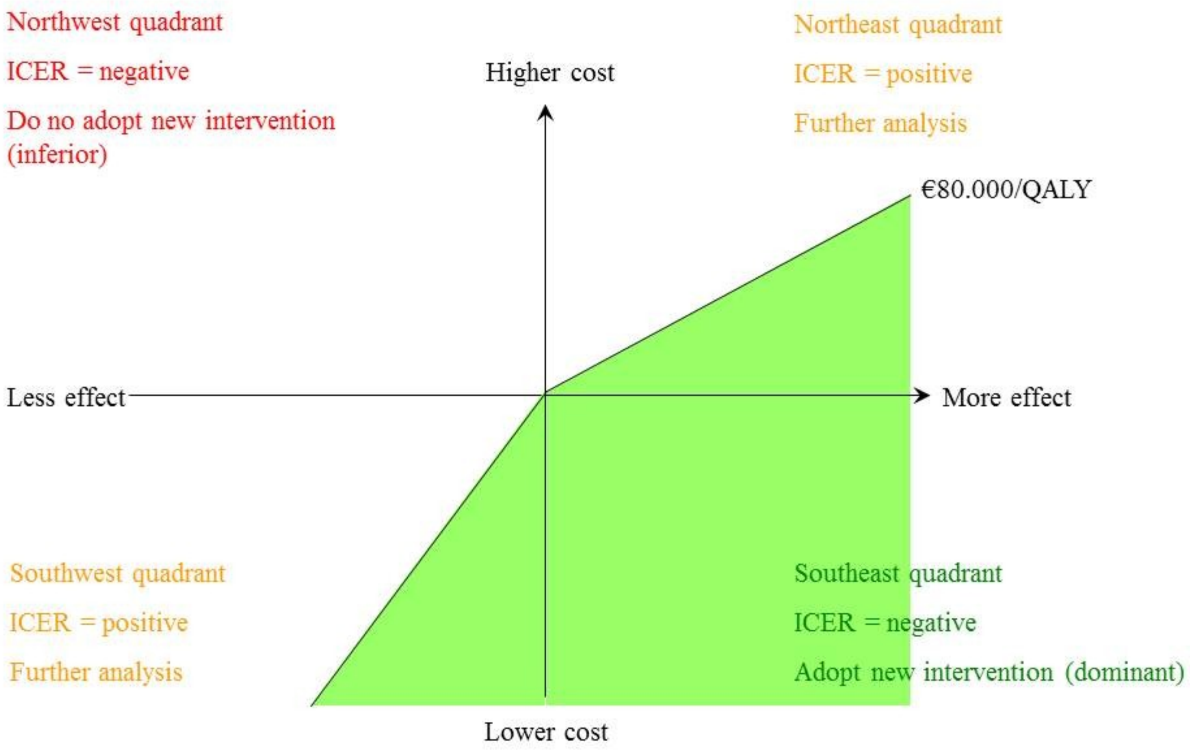

Figure 1. Simplified cost-effectiveness plane [10]

References

1. Drummond MF, O'Brien BJ, Sculpher MJ, Thorrance GW, Stoddart GL. Methods for the economic evaluation of health care programmes. 3rd revised ed. ed. Oxford: Oxford University Press; 2005.
2. Evers SM, Wolfs C, van Heugten C. Economische evaluatie van neuropsychologische behandeling.
3. Drummond MF. Methods for the economic evaluation of health care programmes. 2nd ed. ed. Oxford: Oxford University Press; 1997.
4. Noben C, de Rijk A, Nijhuis F, Evers S. Self-report methods to assess costs: how valid are they? 9th Annual Meeting of Health Technology Assessment International (HTAi); 2012; Bilbao, Spain: Gaceta Sanitaria; 26; 2012.
5. Goossens ME, Rutten van Molken MP, Vlaeyen JW, van der Linden SM. The cost diary: a method to measure direct and indirect costs in cost-effectiveness research. Journal of Clinical Epidemiology. 2000;53(7):688-695.

6. Hakkaart-van Roijen L, Tan SS, Bouwmans CAM. Handleiding voor kostenonderzoek, methoden en standaard kostprijzen voor economische evaluaties in de gezondheidszorg. Geactualiseerde versie 2010 ed: College voor zorgverzekeringen; 2010.
7. Stinnett AA, Mullahy J. Net health benefits: a new framework for the analysis of uncertainty in cost-effectiveness analysis. Medical Decision Making. 1998;18(2 Suppl):S68-80.
8. Raad voor de Volksgezondheid en Zorg. Zinnige en duurzame zorg. Zoetermeer; 2006.
9. Glick HA, Doshi JA, Sonnad SS, Polsky D. Economic Evaluation in Clinical Trials. New York: Oxford University Press; 2007.
10. Evers S, Wolfs C, Van Heugten C. Economische evaluatie van neuropsychologische behandeling. In: Ponds R, van Heugten C, Fasotti L, Wekking E, editors. Neuropsychologische Behandeling. Amsterdam: Boom Uitgeverij; 2010. p. 115-140.
