# Supplementary material for: Cost-Effectiveness and Cost-Utility of Internet-Based Computer Tailoring for Smoking Cessation
Source: J Med Internet Res. 2013 Mar 14;15(3):e57. doi: 10.2196/jmir.2059 (PMC3636293; doi:10.2196/jmir.2059)
Supplement: Supplementary file 2 [file jmir_v15i3e57_app2.pdf]

Multimedia appendix 2. A tailored smoking cessation advice for a participant who reported to still be smoking at six-month follow-up and whose self-efficacy to quit has decreased since baseline.

Step 3. Have confidence in the success of your quit attempt!

You are now less confident that you will succeed in not smoking than the before. That's a pity, because when you feel more confident that you will succeed, it will become easier for you to quit smoking. Therefore, we will go through those situations again, in which you indicated it will be hard for you not to smoke. And on the next pages, we will once more give you the opportunity to make specific plans to cope with these difficult situations. By making specific plans on how to ensure you will not smoke in these situations, the chances will increase that you will succeed in becoming a non-smoker.

- You are not sure whether you will succeed not to smoke when someone offers you a cigarette. And you are right, it is indeed very hard to say 'no' in such a situation. Perhaps you can re-read the advice we have given you last time. In this advice, we extensively described how you can learn to **say 'no'** in such situations. You will find your previous feedback letter by clicking on the **'my advice'** button in the left menu.
- You indicate that you do not think you will manage not to smoke when you're taking a break. For many smokers, is has become a habit to smoke while taking a break. But why don't you try not to stick around your smoking colleagues, when you often smoke during breaks at work? There are probably many other people who do not smoke and with whom you could have a nice chat.
