# Supplementary material for: Cost-Effectiveness and Cost-Utility of Internet-Based Computer Tailoring for Smoking Cessation
Source: J Med Internet Res. 2013 Mar 14;15(3):e57. doi: 10.2196/jmir.2059 (PMC3636293; doi:10.2196/jmir.2059)
Supplement: Supplementary file 3 [file jmir_v15i3e57_app3.pdf]

CONSORT-EHEALTH Checklist V1.6.2 Report

(based on CONSORT-EHEALTH V1.6), available at [http://tinyurl.com/consort-ehealth-v1-6].

2059

Date completed

1/18/2012 10:09:51

by

Eline Suzanne Smit

Cost-effectiveness and cost-utility of Internet-based computer-tailoring for smoking cessation

TITLE

1a-i) Identify the mode of delivery in the title

Yes. "Cost-effectiveness and cost-utility of Internet-based computer-tailoring for smoking cessation: a trial-based economic evaluation among Dutch adult smokers"

1a-ii) Non-web-based components or important co-interventions in title

Yes. "Cost-effectiveness and cost-utility of Internet-based computer-tailoring for smoking cessation: a trial-based economic evaluation among Dutch adult smokers"

1a-iii) Primary condition or target group in the title

Yes. "Cost-effectiveness and cost-utility of Internet-based computer-tailoring for smoking cessation: a trial-based economic evaluation among Dutch adult smokers"

ABSTRACT

1b-i) Key features/functionalities/components of the intervention and comparator in the METHODS section of the ABSTRACT

Yes. "Objective: To assess the cost-effectiveness and cost-utility of an Internet-based multiple computer tailored smoking cessation program and tailored counseling by practice nurses working in Dutch general practices (MTC) compared with the Internet-based multiple computer tailored program only (MT) and care as usual (UC)."

1b-ii) Level of human involvement in the METHODS section of the ABSTRACT

Yes. "Objective: To assess the cost-effectiveness and cost-utility of an Internet-based multiple computer tailored smoking cessation program and tailored counseling by practice nurses working in Dutch general practices (MTC) compared with the Internet-based multiple computer tailored program only (MT) and care as usual (UC)."

1b-iii) Open vs. closed, web-based (self-assessment) vs. face-to-face assessments in the METHODS section of the ABSTRACT

Yes. "The economic evaluation was embedded in a randomized controlled trial, for which 91 practice nurses recruited 414 eligible smokers."

1b-iv) RESULTS section in abstract must contain use data

Yes.

"Smokers were randomized to receive MTC (N=163), MT (N=132) or UC (N=119). Self-reported cost and quality of life were assessed during a twelve-month follow-up period. Prolonged abstinence and 24-hour and 7-day point prevalence abstinence were assessed at twelve-month follow-up."

Further details on the enrollment, attrition, etc. rates can be found in a manuscript describing the results of the randomized controlled trial (currently under review).

1b-v) CONCLUSIONS/DISCUSSION in abstract for negative trials

Not applicable.

Further details on the clinical effectiveness of the intervention can be found in a manuscript describing the results of the randomized controlled trial (currently under review)..

INTRODUCTION

2a-i) Problem and the type of system/solution

Yes. "A brief advice from a general practitioner for instance, is one of these effective smoking cessation interventions. However, general practitioners and practice nurses often report a lack of time and skills to provide their patients with elaborate smoking cessation advice. Computer tailoring is another behavioral intervention proven to be effective in increasing smoking cessation rates up to twelve months."

"Due to the automatic generation of the tailored feedback and the fact that computer tailored interventions are increasingly delivered online, the integration of an Internet-based computer tailored program in the general practice setting might limit the burden on health professionals and could potentially be time-, and thus cost, saving. Our research team has therefore developed a smoking cessation intervention consisting of Internet-based multiple computer-tailoring and a single tailored counseling session by a practice nurse."

2a-ii) Scientific background, rationale: What is known about the (type of) system

"Despite their proven clinical effectiveness, information about the relative cost-effectiveness of behavioral smoking cessation interventions is limited."

"Therefore, the present study aimed to assess the cost-effectiveness and cost-utility of an Internet-based multiple computer tailored smoking cessation program combined with (MTC) and without (MT) a single tailored counseling session by a practice nurses compared with care as usual (UC)."

METHODS

3a) CONSORT: Description of trial design (such as parallel, factorial) including allocation ratio

Yes. "Therefore, the present study aimed to assess the cost-effectiveness and cost-utility of an Internet-based multiple computer tailored smoking cessation program combined with (MTC) and without (MT) a single tailored counseling session by a practice nurses compared with care as usual (UC)."

3b) CONSORT: Important changes to methods after trial commencement (such as eligibility criteria), with reasons

No; not applicable.

3b-i) Bug fixes, Downtimes, Content Changes

No; not applicable.

4a) CONSORT: Eligibility criteria for participants

Yes. "Respondents were eligible for participation if they smoked, were motivated to quit within six months, were 18 years or older and were able to understand Dutch sufficiently. Moreover, they had to have access to the Internet."

4a-i) Computer / Internet literacy

Yes. "Respondents were eligible for participation if they smoked, were motivated to quit within six months, were 18 years or older and were able to understand Dutch sufficiently. Moreover, they had to have access to the Internet."

4a-ii) Open vs. closed, web-based vs. face-to-face assessments:

Yes. "From May 2009 till June 2010, smokers (N=414) were recruited by practice nurses (N=91) working in Dutch general practices. To aid practice nurses in the recruitment, several recruitment materials were provided (e.g. desk displays, posters and business cards)."
"Interested smokers could sign up for the study through their practice nurse or on the study website, where they were informed that the study was financed by the Dutch Cancer Society and conducted by researchers from Maastricht University in cooperation with the Dutch Expert Center on Tobacco Control (STIVORO). Additionally, the website included information about the objectives of the study, the randomization procedure and the incentive provided when respondents completed all questionnaires, i.e. a €10 voucher. Respondents could choose their own username and password and were informed that no one but the PAS research team was able to retrieve these passwords. As respondents had to report their e-mail address when signing up for the study, respondents with multiple identities were easily identified and removed from further analyses."

4a-iii) Information giving during recruitment

Yes. "Interested smokers could sign up for the study through their practice nurse or on the study website, where they were informed that the study was financed by the Dutch Cancer Society and conducted by researchers from Maastricht University in cooperation with the Dutch Expert Center on Tobacco Control (STIVORO). Additionally, the website included information about the objectives of the study, the randomization procedure and the incentive provided when respondents completed all questionnaires, i.e. a €10 voucher."

4b) CONSORT: Settings and locations where the data were collected

Yes. "From May 2009 till June 2010, smokers (N=414) were recruited by practice nurses (N=91) working in Dutch general practices."
"Intervention costs, health care costs and patient costs were assessed using a three-month retrospective costing questionnaire consisting of open-ended questions."

4b-i) Report if outcomes were (self-)assessed through online questionnaires

Yes. "Intervention costs, health care costs and patient costs were assessed using a three-month retrospective costing questionnaire consisting of open-ended questions."

4b-ii) Report how institutional affiliations are displayed

Yes. "... on the study website, where they were informed that the study was financed by the Dutch Cancer Society and conducted by researchers from Maastricht University in cooperation with the Dutch Expert Center on Tobacco Control (STIVORO)."

5) CONSORT: Describe the interventions for each group with sufficient details to allow replication, including how and when they were actually administered

5-i) Mention names, credential, affiliations of the developers, sponsors, and owners

Yes. "Our research team has therefore developed a smoking cessation intervention consisting of Internet-based multiple computer-tailoring and a single tailored counseling session by a practice nurse." (in introduction section of the manuscript)
"The Internet-based multiple computer tailored smoking cessation program was based on the I Change model and on a previously developed, effective single computer tailored intervention."

5-ii) Describe the history/development process

Yes. "The Internet-based multiple computer tailored smoking cessation program was based on the I Change model and on a previously developed, effective single computer tailored intervention."

5-iii) Revisions and updating

No; not applicable.

5-iv) Quality assurance methods

No; not applicable.

5-v) Ensure replicability by publishing the source code, and/or providing screenshots/screen-capture video, and/or providing flowcharts of the algorithms used

Yes. "An example of a tailored feedback message is provided in appendix 1."

5-vi) Digital preservation

"Interested smokers could sign up for the study through their practice nurse or on the study website (www.persoonlijkstopadvies.nl), ..."

5-vii) Access

"Respondents were able to access their feedback letters in three ways: it was directly made available online, feedback letters were sent to the respondent by email and they could be printed."

5-viii) Mode of delivery, features/functionalities/components of the intervention and comparator, and the theoretical framework

Yes. "The smoking cessation intervention consisted of an Internet-based multiple computer tailored smoking cessation program and tailored counseling by a practice nurse [38].

Internet-based multiple tailored smoking cessation program
The Internet-based multiple computer tailored smoking cessation program was based on the I Change model [39] and on a previously developed, effective single computer tailored intervention [12, 15]. Respondents received a total of four feedback letters: at baseline, two days after the quit date they had set at baseline, after six weeks and after six months. Feedback was personalized and tailored to several respondent characteristics: gender, cognitive variables (attitude, social influence and self efficacy), intention to quit smoking, goal and relapse prevention strategies (action and coping plans), and smoking behavior. Feedback letters were iterative: the second, third and fourth feedback letters did not only concern the respondent's present state, but also referred to changes respondents had made since they were included in the program. Respondents were able to access their feedback letters in three ways: it was directly made available online, feedback letters were sent to the respondent by email and they could be printed. An example of a tailored feedback message is provided in appendix 1."

5-ix) Describe use parameters

Yes. "Respondents received a total of four feedback letters: at baseline, two days after the quit date they had set at baseline, after six weeks and after six months."

5-x) Clarify the level of human involvement

Yes. "The smoking cessation intervention consisted of an Internet-based multiple computer tailored smoking cessation program and tailored counseling by a practice nurse [38].

Tailored counseling by practice nurses
After receiving the first tailored feedback, respondents in the MTC group were prompted to schedule a counseling meeting with their practice nurse within 6 to 8 weeks. They received this counseling session instead of the third tailored feedback letter at six-week follow-up. To assist practice nurses in guiding counseling sessions, they were provided with a counseling protocol. This protocol consisted of three chapters guiding counseling sessions with three different types of respondents: smokers who had quit successfully, smokers who quit but relapsed, and smokers who did not quit yet. The content of the counseling session was developed to be as similar as possible to the content of the computer tailored feedback and was also tailored to the respondents characteristics mentioned above. After six months, practice nurses were instructed to call their patients to ask them about their progress towards permanent cessation and, if needed, to provide them with additional cessation support."

5-xi) Report any prompts/reminders used

Yes. "Respondents were prompted by e-mail to fill in these questionnaires at six-week, six-month and twelve-month follow-up. By clicking on a link provided in this e-mail, respondents could start filling out their next follow-up questionnaire immediately, by logging into the system. One e-mail reminder was sent each time that, one week after receiving the first invitation, a respondent had still not filled out the particular questionnaire he or she was invited to complete."

5-xii) Describe any co-interventions (incl. training/support)

Yes. "The smoking cessation intervention consisted of an Internet-based multiple computer tailored smoking cessation program and tailored counseling by a practice nurse [38].

Tailored counseling by practice nurses

After receiving the first tailored feedback, respondents in the MTC group were prompted to schedule a counseling meeting with their practice nurse within 6 to 8 weeks. They received this counseling session instead of the third tailored feedback letter at six-week follow-up. To assist practice nurses in guiding counseling sessions, they were provided with a counseling protocol. This protocol consisted of three chapters guiding counseling sessions with three different types of respondents: smokers who had quit successfully, smokers who quit but relapsed, and smokers who did not quit yet. The content of the counseling session was developed to be as similar as possible to the content of the computer tailored feedback and was also tailored to the respondents characteristics mentioned above. After six months, practice nurses were instructed to call their patients to ask them about their progress towards permanent cessation and, if needed, to provide them with additional cessation support."

6a) CONSORT: Completely defined pre-specified primary and secondary outcome measures, including how and when they were assessed

Yes. "The primary outcome measure used in the cost-effectiveness analysis (CEA) was prolonged abstinence at twelve-month follow-up, assessed by one item asking whether the respondent had refrained from smoking since the previous measurement at six-month follow-up (i.e. abstinence for at least six months) (1=no; 2=yes). Secondary outcome measures were seven day point prevalence abstinence, assessed by one item asking whether the respondent had refrained from smoking during the past seven days (1=no; 2=yes) and addiction level, measured by the abbreviated Fagerström Test for Nicotine Dependence (0=not addicted; 10=highly addicted) [42]. Self-reported abstinence at twelve-month follow-up was cotinine validated using a saliva swap test."

"The primary outcomes measure for the cost-utility analysis (CUA) was quality of life, assessed in terms of Quality Adjusted Life Years (QALYs) based on the EuroQol (EQ-5D) [34, 45]."

6a-i) Online questionnaires: describe if they were validated for online use and apply CHERRIES items to describe how the questionnaires were designed/deployed

No; not applicable for the present manuscript presenting an economic evaluation study. Further details on the questionnaires used can be found in another manuscript:

Smit, E.S., de Vries, H., Hoving, C. (2010). The PAS study: A Randomized Controlled Trial evaluating the effectiveness of a web-based multiple tailored smoking cessation programme and tailored counseling by practice nurses, Contemporary Clinical Trials, 31, 251-258. DOI: 10.1016/j.cct.2010.03.001.

6a-ii) Describe whether and how "use" (including intensity of use/dosage) was defined/measured/monitored

No; not applicable for the present manuscript presenting an economic evaluation study. Further details on the definition of 'use' can be found in a manuscript descirbing the results of the randomized controlled trial (currently under review).

6a-iii) Describe whether, how, and when qualitative feedback from participants was obtained

No; not applicable for the present manuscript.

6b) CONSORT: Any changes to trial outcomes after the trial commenced, with reasons

No; not applicable.

7a) CONSORT: How sample size was determined

7a-i) Describe whether and how expected attrition was taken into account when calculating the sample size

No; not applicable for the present manuscript presenting an economic evaluation study. Further details on the definition of 'use' can be found in a manuscript descirbing the results of the randomized controlled trial (currently under review).

7b) CONSORT: When applicable, explanation of any interim analyses and stopping guidelines

No; not applicable.

8a) CONSORT: Method used to generate the random allocation sequence

Yes. "After providing informed consent, participants were randomized into one of the two intervention groups, MTC (N=163) or MT (N=132), or in the UC control group (N=119). Randomization took place at respondent level by means of a computer software randomization device."

8b) CONSORT: Type of randomisation; details of any restriction (such as blocking and block size)

Yes. "After providing informed consent, participants were randomized into one of the two intervention groups, MTC (N=163) or MT (N=132), or in the UC control group (N=119). Randomization took place at respondent level by means of a computer software randomization device."; not applicable.

9) CONSORT: Mechanism used to implement the random allocation sequence (such as sequentially numbered containers), describing any steps taken to conceal the sequence until interventions were assigned

Yes. "After providing informed consent, participants were randomized into one of the two intervention groups, MTC (N=163) or MT (N=132), or in the UC control group (N=119). Randomization took place at respondent level by means of a computer software randomization device."

10) CONSORT: Who generated the random allocation sequence, who enrolled participants, and who assigned participants to interventions

Yes. "After providing informed consent, participants were randomized into one of the two intervention groups, MTC (N=163) or MT (N=132), or in the UC control group (N=119). Randomization took place at respondent level by means of a computer software randomization device."

11a) CONSORT: Blinding - If done, who was blinded after assignment to interventions (for example, participants, care providers, those assessing outcomes) and how

11a-i) Specify who was blinded, and who wasn't

Yes. "Blinding of respondents was not possible as they had to take notice of whether or not they were receiving tailored feedback."

11a-ii) Discuss e.g., whether participants knew which intervention was the "intervention of interest" and which one was the "comparator"

"Blinding of respondents was not possible as they had to take notice of whether or not they were receiving tailored feedback."

11b) CONSORT: If relevant, description of the similarity of interventions

No; not applicable.

12a) CONSORT: Statistical methods used to compare groups for primary and secondary outcomes

Yes. "All analyses were conducted according to the intention-to-treat principle. Missing data for costs, EQ-5D items, overall tobacco consumption and addiction level were replaced by mean imputation, using respondents' scores on the previous and next measurement. When mean imputation was not possible due to missing data on multiple measurement points, missing data were replaced using the last observation carried forward (preferred choice) or next observation carried backward method. Missing data for smoking abstinence were replaced using a negative scenario, whereby respondents lost to follow-up were considered still smoking.

Baseline comparability of the three study groups
To investigate the comparability of the three groups with regard to demographics, baseline values of outcomes and healthcare-related costs over the last three months, one-way analyses of variance (ANOVA) with Tukey's posthoc tests and Chi-square tests were conducted. To determine whether selective drop-out had occurred, a comparison was made between those lost to follow-up and those who remained in the study after twelve months using two-sided t-tests and Chi-square tests.

Annual costs and effects
The three groups were compared with regard to their mean annual costs using non-parametric bootstrapping (5,000 times) with 95% confidence intervals in percentiles. To compare the three groups with regard to the mean effect assessed twelve months after baseline, one-way analyses of variance with Tukey's posthoc tests and Chi-square tests were conducted.

Cost-effectiveness and cost-utility analyses
First, incremental costs and effects were calculated for each of the three treatments studied. Subsequently, NMBs were calculated enabling us to compare the three groups directly with each other regarding their cost-effectiveness and cost-utility. Using a range of thresholds for the willingness to pay, the likelihood was calculated that each treatment would be most efficient.

Uncertainty analysis
Sampling uncertainty around the estimates of cost-effectiveness and cost-utility was taken into account using non-parametric bootstrap re-sampling techniques. To deal with the uncertainty of parameter estimates from the primary analyses, a sensitivity analysis was conducted. In primary and secondary analyses patient costs (i.e. traveling and time costs) were not valued in monetary costs but considered as reflected in participants' reported quality of life. However, as patient costs can be considered directly related to the treatment received it could be argued that these costs should be included as part of the program cost. Therefore, we tested whether an increase in program costs as a result of patient costs' monetary valuation would lead to a change in results. For the MTC group, the inclusion of patient costs meant an increase in program costs from €57.70 to €141.89 and for the MT group this meant an increase in program costs from €7.70 to €82.24.
Bootstrap analyses were conducted using Microsoft Office Excel 2003. All other analyses were conducted using SPSS 17.0."

12a-i) Imputation techniques to deal with attrition / missing values

Yes. "Missing data for costs, EQ-5D items, overall tobacco consumption and addiction level were replaced by mean imputation, using respondents' scores on the previous and next measurement. When mean imputation was not possible due to missing data on multiple measurement points, missing data were replaced using the last observation carried forward (preferred choice) or next observation carried backward method. Missing data for smoking abstinence were replaced using a negative scenario, whereby respondents lost to follow-up were considered still smoking."

12b) CONSORT: Methods for additional analyses, such as subgroup analyses and adjusted analyses

Yes. "Uncertainty analysis
Sampling uncertainty around the estimates of cost-effectiveness and cost-utility was taken into account using non-parametric bootstrap re-sampling techniques. To deal with the uncertainty of parameter estimates from the primary analyses, a sensitivity analysis was conducted. In primary and secondary analyses patient costs (i.e. traveling and time costs) were not valued in monetary costs but considered as reflected in participants' reported quality of life. However, as patient costs can be considered directly related to the treatment received it could be argued that these costs should be included as part of the program cost. Therefore, we tested whether an increase in program costs as a result of patient costs' monetary valuation would lead to a change in results. For the MTC group, the inclusion of patient costs meant an increase in program costs from €57.70 to €141.89 and for the MT group this meant an increase in program costs from €7.70 to €82.24.
Bootstrap analyses were conducted using Microsoft Office Excel 2003. All other analyses were conducted using SPSS 17.0."

RESULTS

13a) CONSORT: For each group, the numbers of participants who were randomly assigned, received intended treatment, and were analysed for the primary outcome

Yes. "Of the 414 respondents who were eligible for participation, 163 were randomized into the MTC group, 132 into the MT group and 119 into the UC group."

13b) CONSORT: For each group, losses and exclusions after randomisation, together with reasons

"After twelve months, 231 (55.8%) of the 414 respondents could be followed up." Further details on attrition, including a CONSORT flow diagram, can be found in a manuscript descirbing the results of the randomized controlled trial (currently under review).

13b-i) Attrition diagram

No; not applicable for the present manuscript presenting an economic evaluation study. Further details on attrition, including a CONSORT flow diagram, can be found in a manuscript descirbing the results of the randomized controlled trial (currently under review).

14a) CONSORT: Dates defining the periods of recruitment and follow-up

Yes. "From May 2009 till June 2010, smokers (N=414) were recruited by practice nurses (N=91) working in Dutch general practices."
"Respondents were prompted by e-mail to fill in these questionnaires at six-week, six-month and twelve-month follow-up."

14a-i) Indicate if critical "secular events" fell into the study period

No; not applicable.

14b) CONSORT: Why the trial ended or was stopped (early)

No; not applicable.

15) CONSORT: A table showing baseline demographic and clinical characteristics for each group

Yes; table 1.

15-i) Report demographics associated with digital divide issues

Yes. "No baseline differences were found (Table 1)."
Table 1 also includes data on educational level.

16a) CONSORT: For each group, number of participants (denominator) included in each analysis and whether the analysis was by original assigned groups

16-i) Report multiple "denominators" and provide definitions

Yes. "Of the 414 respondents who were eligible for participation, 163 were randomized into the MTC group, 132 into the MT group and 119 into the UC group."
"After twelve months, 231 (55.8%) of the 414 respondents could be followed up." Further details on attrition, including a CONSORT flow diagram, can be found in a manuscript descirbing the results of the randomized controlled trial (currently under review).

16-ii) Primary analysis should be intent-to-treat

Yes. "All analyses were conducted according to the intention-to-treat principle." (methods section of the manuscript).

17a) CONSORT: For each primary and secondary outcome, results for each group, and the estimated effect size and its precision (such as 95% confidence interval)

Results of economic evaluation studies are presented differently from results of 'standard' trial studies. The results of the study presented are described as follows:

"Cost-effectiveness analyses

Table 3 shows that, compared with UC, €5,100 has to be paid in the MT group for each additional respondent being abstinent. For respondents in the MTC group costs were higher, while effects were lower than in the UC and MT groups, As a result, MTC was dominated by the other two treatments. The CEA showed that until a threshold value for the WTP of €5,100 per abstinent respondent, UC was most probably the most efficient treatment. From a WTP of €5,100 or higher, however, MT was most probably most cost-effective (Table 4). With the accepted Dutch cut off point of €18,000 per QALY for preventive interventions [36], MT would thus be the most preferable treatment. These results are visually displayed in the cost-effectiveness acceptability curve (CEAC), showing the probability of each treatment being preferable to the other two treatments for varying levels of the WTP per additional abstinent participant (Figure 2). Sensitivity analyses supported these results (Table 4).

Results from secondary analyses showed that concerning seven-day point prevalence abstinence, a high probability was found (i.e. 88%, with a WTP of €18,000 per abstinent respondent) that MT was the most cost-effective treatment. Regarding the level of addiction, however, it was most probable that MT would be least efficient (Table 4).

Cost-utility analyses

With regard to QALYs gained, results were somewhat different. As is shown in table 3, MT was dominated, as this treatment was both more expensive and less effective than UC. MTC was more expensive, but also more successful than UC and MT, which resulted in an incremental cost of €40,300 per QALY gained when comparing MTC with UC. Comparing MTC with MT resulted in an incremental cost of €18,367 per QALY.

The CUA showed that, with a WTP of €18,000 per abstinent respondent, UC would probably (i.e. 64%) be the most efficient treatment (Table 4). While decreasing this threshold value to €0 led to an increased probability that UC would be most efficient, increasing this threshold led to a lower probability of UC being most preferable. With a WTP of almost €40,000, UC and MTC would be equally preferable. These results are further illustrated in the cost-utility acceptability curve (CUAC) (Figure 3). Sensitivity analyses showed similar results (Table 4)."

17a-i) Presentation of process outcomes such as metrics of use and intensity of use

No; not applicable for the present manuscript.

17b) CONSORT: For binary outcomes, presentation of both absolute and relative effect sizes is recommended

Results of economic evaluation studies are presented differently from results of 'standard' trial studies. The results of the study presented are described as follows:

"Cost-effectiveness analyses

Table 3 shows that, compared with UC, €5,100 has to be paid in the MT group for each additional respondent being abstinent. For respondents in the MTC group costs were higher, while effects were lower than in the UC and MT groups, As a result, MTC was dominated by the other two treatments. The CEA showed that until a threshold value for the WTP of €5,100 per abstinent respondent, UC was most probably the most efficient treatment. From a WTP of €5,100 or higher, however, MT was most probably most cost-effective (Table 4). With the accepted Dutch cut off point of €18,000 per QALY for preventive interventions [36], MT would thus be the most preferable treatment. These results are visually displayed in the cost-effectiveness acceptability curve (CEAC), showing the probability of each treatment being preferable to the other two treatments for varying levels of the WTP per additional abstinent participant (Figure 2). Sensitivity analyses supported these results (Table 4).

Results from secondary analyses showed that concerning seven-day point prevalence abstinence, a high probability was found (i.e. 88%, with a WTP of €18,000 per abstinent respondent) that MT was the most cost-effective treatment. Regarding the level of addiction, however, it was most probable that MT would be least efficient (Table 4).

Cost-utility analyses

With regard to QALYs gained, results were somewhat different. As is shown in table 3, MT was dominated, as this treatment was both more expensive and less effective than UC. MTC was more expensive, but also more successful than UC and MT, which resulted in an incremental cost of €40,300 per QALY gained when comparing MTC with UC. Comparing MTC with MT resulted in an incremental cost of €18,367 per QALY.

The CUA showed that, with a WTP of €18,000 per abstinent respondent, UC would probably (i.e. 64%) be the most efficient treatment (Table 4). While decreasing this threshold value to €0 led to an increased probability that UC would be most efficient, increasing this threshold led to a lower probability of UC being most preferable. With a WTP of almost €40,000, UC and MTC would be equally preferable. These results are further illustrated in the cost-utility acceptability curve (CUAC) (Figure 3). Sensitivity analyses showed similar results (Table 4)."

18) CONSORT: Results of any other analyses performed, including subgroup analyses and adjusted analyses, distinguishing pre-specified from exploratory

Yes. Cost-effectiveness: "Sensitivity analyses supported these results (Table 4)."

"Results from secondary analyses showed that concerning seven-day point prevalence abstinence, a high probability was found (i.e. 88%, with a WTP of €18,000 per abstinent respondent) that MT was the most cost-effective treatment. Regarding the level of addiction, however, it was most probable that MT would be least efficient (Table 4)."

Cost-utility: "Sensitivity analyses showed similar results (Table 4)."

18-i) Subgroup analysis of comparing only users

No; not applicable for the present manuscript.

19) CONSORT: All important harms or unintended effects in each group

No; not applicable.

19-i) Include privacy breaches, technical problems

No; not applicable.

19-ii) Include qualitative feedback from participants or observations from staff/researchers

No; not applicable.

DISCUSSION

20) CONSORT: Trial limitations, addressing sources of potential bias, imprecision, multiplicity of analyses

20-i) Typical limitations in ehealth trials

Yes; in a strengths and limitations section.
"The present study aimed to contribute to the literature by examining the cost-effectiveness and cost-utility of an Internet-based smoking cessation intervention, something which has, to our knowledge, not been done before. In the present study, besides generic quality of life, disease-specific effects of the intervention, i.e. smoking abstinence, were taken into account. To facilitate the comparison of the cost-effectiveness of interventions targeting different diseases, effects are usually assessed in terms of quality of life. However, to compare smoking cessation interventions more specifically, disease-specific effect measures might be more informative. That in the present study both effect measures were included can thus be an important strength. Nevertheless, the present study also had its limitations. First, it suffered from relatively high drop-out rates. High rates of attrition seem to be inherent to many Internet-based interventions and drop-out rates of 44% are not extraordinary when compared to other Internet-based studies [21, 55-57]. As a consequence, however, there was not sufficient power for us to conduct a complete-case analysis as part of the sensitivity analyses. Secondly, as we expected higher attrition rates in the MTC group, at baseline slightly more respondents were randomized into this intervention group. Attrition rates, however, appeared to be similar among the groups, resulting in a skewed distribution of respondents with 163 respondents in the MTC group, 132 in the MT group and 119 in the UC group. We do no expect that this has biased our results. In fact, our finding of no selective attrition can be considered valuable in the design of future trials."

21) CONSORT: Generalisability (external validity, applicability) of the trial findings

21-i) Generalizability to other populations

Yes. "While a willingness to pay of €18,000 per QALY is an accepted Dutch cut-off point, no such cut-off point exist with regard to abstinence rates."

21-ii) Discuss if there were elements in the RCT that would be different in a routine application setting

No; not applicable for this economic evaluation manuscript.

22) CONSORT: Interpretation consistent with results, balancing benefits and harms, and considering other relevant evidence

22-i) Restate study questions and summarize the answers suggested by the data, starting with primary outcomes and process outcomes (use)

Yes. "To our current knowledge, this was the first study to determine the cost-effectiveness and cost-utility of an Internet-based smoking cessation program with and without counseling by a practice nurse. The results presented suggest that respondents who received the Internet-based multiple computer tailored program and tailored counseling by their practice nurse reported significantly more annual healthcare related costs than respondents who received care as usual."
"Furthermore, the present study showed that the Internet-based multiple computer tailored smoking cessation program would probably be the most cost-effective of the three treatments under study."
"When it concerned cost-utilities, the results presented suggest that care as usual would probably be the most preferable of the treatments studied."

22-ii) Highlight unanswered new questions, suggest future research

Yes. "To enable the interpretation of the incremental costs per abstinent participant, future research should aim at identifying an acceptable cut-off point for the willingness to pay per abstinent participant."

Other information

23) CONSORT: Registration number and name of trial registry

Yes. "Trial ID number Dutch Trial Register NTR1351." (abstract and methods section)

24) CONSORT: Where the full trial protocol can be accessed, if available

Yes. "A more detailed description of the study design has been published elsewhere [38]."

25) CONSORT: Sources of funding and other support (such as supply of drugs), role of funders

Yes. "The study was funded by the Dutch Cancer Society (UM 2007-3834)."

X26-i) Comment on ethics committee approval

Yes. "The trial design was approved by the Medical Ethics Committee of Maastricht University and the University Hospital Maastricht (MEC 08-3-037; NL22692.068.08), and is registered with the Dutch Trial Register (NTR1351)."

x26-ii) Outline informed consent procedures

Yes. "on the study website (www.persoonlijkstopadvies.nl), where they were informed that the study was financed by the Dutch Cancer Society and conducted by researchers from Maastricht University in cooperation with the Dutch Expert Center on Tobacco Control (STIVORO). Additionally, the website included information about the objectives of the study, the randomization procedure and the incentive provided when respondents completed all questionnaires, i.e. a €10 voucher. Respondents could choose their own username and password and were informed that no one but the PAS research team was able to retrieve these passwords."
"After providing informed consent, participants were randomized ..."

X26-iii) Safety and security procedures

Yes. "Respondents could choose their own username and password and were informed that no one but the PAS research team was able to retrieve these passwords."

X27-i) State the relation of the study team towards the system being evaluated

Yes. "Hein de Vries is scientific director of Vision2Health, a company that licenses evidence-based innovative computer-tailored health communication tools."
